# Comparing the Printability, Biological and Physicochemical Properties of Bio-Based Photo-Crosslinkable Hydrogels

**DOI:** 10.3390/polym17212867

**Published:** 2025-10-28

**Authors:** Ane García-García, Unai Silván, Leyre Pérez-Álvarez, Senentxu Lanceros

**Affiliations:** 1 Innovative Macromolecular Materials Group (Imacromat), Physical Chemistry Department, Faculty of Science and Technology, University of the Basque Country UPV/EHU, 48940 Leioa, Spain; ane.garcia@bcmaterials.net; 2 BCMaterials, Basque Center for Materials, Applications and Nanostructures, UPV/EHU Science Park, 48940 Leioa, Spain; unai.silvan@bcmaterials.net (U.S.); senentxu.lanceros@bcmaterials.net (S.L.); 3 Ikerbasque, Basque Foundation for Science, 48009 Bilbao, Spain

**Keywords:** photo-crosslinkable hydrogels, extrusion-based 3D printing, methacrylated alginate, methacrylated chitosan, methacrylated gelatin

## Abstract

Bio-based photo-crosslinkable hydrogels are used in tissue engineering as three-dimensional printable scaffolds due to their functional and biological similarities with the extracellular matrix (ECM). In this work, emerging bioink candidates such as chitosan, alginate and gelatin-based photo-crosslinkable hydrogel were developed using extrusion-based 3D printing to establish a better understanding of their applicability. The polymers were methacrylated by the same methacrylation reaction pathway, which enabled successful light-induced 3D printing. Morphology, swelling (6–40%), mechanical (Young’s modulus, 0.1–0.5 KPa) and rheological properties (300–1000 Pa), degradation kinetics (10->60 days) and printability of the gels were also characterized in identical conditions for the first time. 3D-printability results indicated that methacrylated gelatin enhanced printability, shape fidelity and integrity of printed structures compared to methacrylated alginate, which presents structural instability and poorer printing control due to its low crosslink density. Moreover, cell attachment and Live/Dead assays using bone marrow-derived mesenchymal stem cells (BM-MSCs) showed that all formulations have good biocompatibility for use as scaffolds. Specifically, gelatin-based hydrogels showed a higher level of BM-MSCs attachment and spreading than the other types of hydrogels. Overall, our results suggest that the hydrogels based on these three biopolymers present good potential as a biomaterial for light-induced extrusion-based 3D printing.

## 1. Introduction

The development of materials that provide a three-dimensional environment capable of structural and biochemical support for the tissue, promoting cell proliferation and enhancing cell properties, is crucial for tissue engineering applications [1]. In this regard, 3D printing is identified as a promising technology for the precise fabrication of three-dimensional (3D) scaffolds.

Amongst the range of printing technologies, extrusion-based 3D printing has garnered significant attention due to its simplicity, scalability [2], cost-effectiveness, accessibility and the lack of energy-intensive sources, such as lasers, which could affect the cells [3].

Hydrogels are one of the most promising classes of ink for creating 3D-printed scaffolds, as they possess a high structural and functional similarity to the extracellular matrix [4]. Meanwhile, hydrogel networks present good biocompatibility, biodegradability and high-water absorption capability. Furthermore, hydrogels can be easily modified and dynamically adapted to the changing needs of the culture of different cell types [1,5]. Among the different types of hydrogels, photo-crosslinkable hydrogels enable the fabrication of highly complex and precise 3D structures, since light controls the gelation process spatially and temporally. In addition, photo-crosslinking is a rapid method that works under mild conditions, ensuring the preservation of sensitive biological components such as proteins and cells [6].

Polysaccharides and polymers of peptidic origin are commonly used in the development of printable hydrogels [7]. Polysaccharides are a group of complex polymers isolated from a wide range of renewable resources, including plants, animals, and microbes [8]. This is the case of alginate, which is derived from diverse resources, such as the cell walls of brown seaweed or bacteria and is one of the most widely studied natural polymers [8]. Alginate is composed of two homopolymeric blocks, 1,4-β-D-mannuronic acid (M) and 1,4-α-L-guluronic acid (G) [9] and is typically studied for biomedical applications due to its good biocompatibility, solubility, porosity, hydrophilicity and injectability [7]. Another polysaccharide of natural origin that is commonly used in the form of a hydrogel in tissue engineering is chitosan, which is composed of D-glucosamine and N-acetyl glucosamine units linked by β-glycosidic bonds (1-4). This polymer exhibits excellent antimicrobial and antioxidant activity, mucoadhesivity, biodegradability and high biological compatibility [10,11,12,13]. As a bio-ink for 3D-printing, chitosan has demonstrated stability under physiological conditions, appropriate viscosity, and ability to support effective cell proliferation and differentiation [14]. Regarding natural polymers of peptidic origin, they are widely found in living organisms and generally have specific bioactive activities (including antibacterial, anti-inflammatory, tissue and cell adhesion, promotion of cell proliferation and migration, etc.) [7]. Among them, gelatin is a natural polymer derived from the hydrolyzation of collagen, the major component of cartilage, bone, skin, connective tissue and the extracellular matrix in animals, which presents biocompatibility, biodegradability, low antigenicity, easy processing, abundant availability in nature and cost-effectiveness [15,16]. Due to these valuable characteristics, gelatin is considered an ideal material that can be easily adapted as a bio-ink for 3D printing [17,18,19,20].

In recent years, hydrogels derived from these three biopolymers have emerged as among the most versatile and widely used materials in 3D bio-printing. These natural polymers have been adapted for use in a variety of additive manufacturing techniques, including extrusion-based bio-printing and digital light processing (DLP); however, it remains a challenge to print high-resolution constructs with adequate functionality [21,22,23,24].

In the case of alginate, ionotropic gelation of unmodified alginate is the most widely explored strategy for extrusion-based bio-printing, due to its rapid ionic crosslinking [25], but it results in low-resolution constructs. However, more complex formulations of alginate in combination with other biopolymers have shown high printing fidelity and applicability in a wide range of tissues, including bone [17] or skin [26]. Regarding photo-crosslinkable alginate, the direct 3D extrusion-based photo-printing of concentrated solutions of methacrylated alginate proves to be inefficient in the absence of external crosslinking agents [27]. However, Gharacheh et al. enhanced the printing quality of methacrylated alginate, together with the osteogenic differentiation of hMSCs cells, reducing the concentration of the biopolymer and incorporating human allograft bone particles [27].

On the other hand, gelatin, which presents good biodegradability and cell adhesiveness, has also been shown to be a suitable candidate for cell encapsulation and printing [18]. Specifically, methacrylated gelatin is commonly employed as bioink in extrusion-based photo-printing. Methacrylated gelatin was 3D-printed with high fidelity via extrusion with photo-crosslinking (12.5 mW/cm^2^) and successfully tested in vivo in a rat condyle defect [28].

Chitosan-based hydrogels, due to their antimicrobial and anti-inflammatory properties [19], have been widely incorporated into bio-printed scaffolds for applications in skin [19] and nerve regeneration [29]. We previously demonstrated the successful extrusion-based printability, biocompatibility and antimicrobial properties of slightly photo-cured methacrylated chitosan scaffolds [14]. However, photo-curing conditions led to poor mechanical properties, which could affect their long-term stability and applicability.

Taking the above into consideration, this study compares 3D printable scaffolds based on photo-crosslinkable alginate, chitosan and gelatin. In recent years, studies on these systems have proliferated, but there is no common criterion for methacrylation conditions, ink formulations, or printing conditions—the main parameters that serve as the basis for comparison. In this respect, the hydrogels were synthesized via photo-crosslinking after methacrylation of the biopolymers, using the same reaction conditions. They were also characterized under identical conditions in terms of morphology, swelling, degradation, rheological and mechanical stability, properties that are highly influenced by hydrogel composition. Additionally, the adhesion properties and cytocompatibility of hydrogels were synchronously and identically characterized in vitro to determine their potential to support cell attachment, proliferation, and viability as biomaterials. This work hypothesizes that by comparatively analyzing the individual properties of these commonly used biocompatible and printable hydrogels, we can provide a practical framework for the selection of suitable bio-inks for specific applications. In addition, these results add fundamental knowledge for the development of functional 3D-printed scaffolds suitable for regenerative medicine applications.

## 2. Materials and Methods

### 2.1. Materials

The synthesis of the photo-crosslinked hydrogels was performed by modifying sodium alginate (Sigma-Aldrich, Darmstadt, Germany, W201502, M_w_ = 1.1 × 10^5^ g/mol), chitosan from crab shells (Sigma-Aldrich, Darmstadt, Germany, 48165, M_w_ = 8.7 × 10^5^ ± 4 × 10^4^ g/mol) and gelatin from porcine skin type A (Sigma-Aldrich, Darmstadt, Germany, G1890, M_w_ = 1.7 × 10^5^ g/mol) with methacrylic anhydride (Sigma-Aldrich, Darmstadt, Germany, 276685). In addition, the photoinitiator lithium phenyl-2,4,6-trimethylbenzoylphosphinate (LAP, Sigma-Aldrich, Darmstadt, Germany, 900889), acetic acid (Sigma Aldrich, Darmstadt, Germany, 33209 for analysis, ≥99.8%), potassium phosphate monobasic (Acros Organics, Geel, Belgium, 447670010, 99%, for analysis), sodium phosphate dibasic (Acros Organics, Geel, Belgium, 448140010, 98%, extra pure), sodium hydroxide (Panreac, Castellar del Valles, Spain, 141687.1211, pure, pharma grade), ethanol (Panreac, Castellar del Valles, Spain,141086.0716, absolute), glutaraldehyde aqueous solution (Sigma-Aldrich, Darmstadt, Germany, 354400, 25%), (3-aminopropyl)triethoxysilane (APTES, Sigma-Aldrich, Darmstadt, Germany, 440140), 3-(trimethylsilyl)-1-propanesulfonic acid sodium salt (TSP, Sigma-Aldrich, Darmstadt, Germany, 178837, 97%), deuterium oxide (Sigma-Aldrich, Darmstadt, Germany, 151882, 99 atom D), acetic acid-d_4_ (Sigma-Aldrich, Darmstadt, Germany, 151785, ≥99.5% atom D), and Human Bone Marrow-derived Mesenchymal Stem Cells (BM-MSCs) were purchased by Cell Lines Service (Heidelberg, Alemania, Catalog number CLS-300665). Dulbecco’s Modified Eagle Medium (DMEM, Gibco, Waltham, MA, USA, 30030), fetal bovine serum (FBS, Sigma-Aldrich, SV30160.03), formaldehyde (Sigma-Aldrich, Darmstadt, Germany, 50-00-0), penicillin and streptomycin antibiotics (Gibco, 15140-122), phosphate-buffered saline (PBS, Gibco, 10010-023), NucBlue^TM^ Live Cell Stain ReadyProbes^TM^ Reagent (Thermo Fisher, Waltham, MA, USA, R37605), and LIVE/DEAD^TM^ Cell Imaging kit (Thermo Fisher, Waltham, MA, USA, R37601) were used.

### 2.2. Preparation of Photo-Crosslinkable Hydrogels

#### 2.2.1. Methacrylation of the Biopolymers

The modification of chitosan was carried out following the method proposed by Kolawole et al. [30] with slight modifications. Methacrylic anhydride (3.1 mL) was added to a 1.5% (*w*/*v*) chitosan solution (0.5% (*v*/*v*) in acetic acid solution and kept stirring at 45 °C for 24 h. The resulting product was then purified through dialysis (12–14 kDa membranes) over 5 days and lyophilized at −50 °C and 0.1 mBar.

For the modification of alginate, the procedure of Chou et al. was followed [31]. Briefly, a 2% (*w*/*v*) solution of sodium alginate in distilled water was prepared. Then, a 20% molar excess of methacrylic anhydride was added to the solution while maintaining the pH at 7.5 using a solution of NaOH 5 M, with constant magnetic stirring for 2 h. Then, the final solution was maintained at 4–8 °C for 24 h, and the reaction was washed with 200 mL of ethanol (30 min × 5 times). The product obtained was dissolved in 60 mL of distilled water, purified via dialysis (3.5 kDa membranes) against sterile water for 5 days and lyophilized at −50 °C and 0.1 mBar.

The modification of gelatin took place according to the procedure of Bektas et al. [32]. Firstly, 10 mL of methacrylic anhydride was added to a 10% (*w*/*v*) solution of gelatin from porcine skin type A (PBS, 50 °C). The obtained product was purified via dialysis (12–14 kDa membranes) against sterile water at 40 °C for 5 days and lyophilized at −50 °C and 0.1 mBar.

#### 2.2.2. Synthesis of Photo-Crosslinkable Hydrogels

For the synthesis of the photo-crosslinked hydrogels of chitosan, a solution of methacrylated chitosan (CHIMe) 1.5% (*w*/*v*) was prepared in a 0.5% (*v*/*v*) acetic acid solution. Separately, the photoinitiator LAP, in a 0.1% (*w*/*w*) concentration with respect to the chitosan, was dissolved using a negligible volume of the previous acetic acid solution (300 µL approximately). Finally, the hydrogels were photo-crosslinked in situ under UV light LEDs (405 nm, 19 mW/cm^2^) for 30 s. The same procedure was carried out for the photo-crosslinked hydrogels of alginate, but using a solution of methacrylated alginate (AlgMe) 3% (*w*/*v*) in PBS (pH = 7.4), and for the photo-crosslinked hydrogels of gelatin (GelMe), but employing a solution of methacrylated gelatin (GelMe) 8% (*w*/*v*) prepared in PBS (pH = 7.4).

### 2.3. Nuclear Magnetic Resonance (^1^H-NMR)

The degree of methacrylation of the photo-crosslinked hydrogels was determined by nuclear magnetic resonance spectroscopy, using a Bruker Avance (Billerica, MA, USA, 500 MHz spectrometer. In the case of CHIMe, the spectrum was taken using a concentration of 20 mg/mL of the modified polymer in a 99.5% D_2_O and 0.5% acetic acid-d_4_ at 25 °C. According to literature [30], the methacrylation degree (MD) was calculated based on the ratio of the relative areas of the integration of the protons of the N-acetyl-glucosamine units of chitosan at 2.8–3.9 ppm, and those of the resonance peaks of the methacrylate groups at 5.6–6.0 ppm (Equation (1)).

(1) $$\mathrm{MD}\;\mathrm{CHIMe}\;(\%)=\frac{Integral\;of\;methacrylate\;protons\;at\;5.6-6.0\;ppm/2\;}{Integral\;of\;chitosan\;cycle\;protons\;at\;2.8-3.9\;ppm/6}\times 100$$

For AlgMe, the spectrum was taken using a concentration of 20 mg/mL in D_2_O at 85 °C, in order to move the water signal out of the spectral region of interest [33]. Based on literature and under the assumption that methacrylation only occurs on guluronic (G) units, both the G unit ratio in the alginate chain and the degree of methacrylation were determined using Equations (2) and (3), respectively. The integral values of the anomeric proton signals of the guluronic and mannuronic units at 5 and 4.3 ppm correspond to H_G_ and H_M_. Likewise, H_a_ and H_b_ refer to the integrals of the two vinyl protons of the methacrylic group, located at 5.7 and 6.2 ppm [34].

(2) $$G\;(\%)=\frac{H_{G}}{H_{M}+H_{G}}\times 100$$

(3) $$\mathrm{MD}\;\mathrm{AlgMe}\;(\%)=\frac{\frac{H_{a}+H_{b}}{2}}{H_{G}}\times G$$

Finally, for GelMe, the spectrum was taken using a concentration of 20 mg/mL in D_2_O and at 45 °C. To calculate the degree of methacrylation, the method of internal standard using 3-(trimethylsilyl)-1-propanesulfonic acid sodium salt (TSP) was used. The TSP signal appears at 0 ppm and corresponds to 9 protons (H_TSP_). To calculate the amount of methacrylic units present in the gelatin derivatives, only the integral obtained for the peak with the highest chemical shift (5.6 ppm) was used, which corresponds to one proton (H_M_) [35]. Equation (4) was used to calculate the degree of methacrylation of GelMe.

(4) $$\mathrm{MD}\;\mathrm{GelMe}\;(\%)=\frac{H_{M}}{H_{TSP}}\times \frac{9}{1}\times \frac{n\left(TSP\right)[mmol]}{m\left(gelatin\right)[g]}$$

The shown data corresponds to the average of three samples.

### 2.4. Morphological Characterization

The morphology and the pore size of the hydrogels were studied using a Hitachi (Tokyo, Japan) S-4800 scanning electron microscope (SEM) (150 s, 20 mA, 15 kV). Hydrogels were previously lyophilized (−50 °C and 0.1 mBar) and then coated with gold. Pore size was determined using ImageJ 1.49 software and was the average of 20 diameters in each sample.

### 2.5. Physico-Chemical Characterization

#### 2.5.1. In Vitro Swelling

Lyophilized hydrogels were immersed in phosphate buffer solution (PBS) at pH = 7.4 and 37 °C, and the swelling factor was calculated over time according to Equation (5):

(5) $$\mathrm{Swelling}\;\mathrm{factor}\;=\;\frac{W_{s}-W_{d}}{W_{d}}$$

where W_s_ and W_d_ are the weights of the swollen and dried hydrogels, respectively. The shown data correspond to the average of three samples.

#### 2.5.2. In Vitro Degradation

Fresh hydrogels were immersed in phosphate buffer solution (PBS) at pH = 7.4 and 37 °C. The mass loss over time was monitored as a quantification of the hydrolytic degradation using Equation (6):

(6) $$\mathrm{Mass}\;\mathrm{loss}\;(\%)\;=\;\frac{W_{0}-W_{t}}{W_{t}}\times 100$$

where W_0_ is the weight of the hydrogel at the initial time and W_t_ is the weight at a specific time. Three samples were evaluated for each data point.

Additionally, the sol-fraction was also measured. For this, lyophilized samples were weighed (W_dry1_) and incubated in the corresponding solvent at 37 °C for 24 h. Then, each sample was lyophilized and weighed (W_dry2_). The sol fraction (%) was calculated as follows (Equation (7)) [36]:

(7) $$\mathrm{Sol-fraction}\;(\%)\;=\;\frac{W_{dry1}-W_{dry2}}{W_{dry1}}\times 100$$

#### 2.5.3. Compressive Stress–Strain Test

A compression stress–strain test of hydrogels was performed using a Metrotec (Lezo, Spain) FTM-50 texture analyzer equipped with a 20 N load cell. Measurements were made at a constant rate of 10 mm/min until mechanical failure. Young’s modulus (E) was determined from a linear regression on the stress–strain curves between 10–20% strain. A total of three replicates were analyzed for each sample.

#### 2.5.4. Rheology

Rheological measurements of the photo-crosslinked hydrogels were carried out with a Rheometric Scientific Advanced Rheometric Expansion System (ARES, TA Instruments, Waters Corporation, New Castle, PA, USA) equipped with a 25 mm diameter parallel plate geometry and a 1.5 mm gap. The viscoelastic limit was determined through a shear strain sweep. Subsequently, the storage (G’) and loss (G”) modulus were measured at 25 °C over a frequency range of 0.1 to 500 rad/s, at a fixed strain of 1%.

#### 2.5.5. 3D Printing Quality

Using the CellInk HeartWare designer 2.4 software, a square grid scaffold of 40 × 40 mm was designed with a space between lines of 5 × 5 mm. The printing quality of each hydrogel was evaluated using the CellInk (Gothenburg, Sweeden) printer INKREDIBLE + at optimized conditions, such as a printing speed of 600 mm/min, a pressure of 21, 25 and 18 kPa for CHIMe, AlgMe, and GelMe hydrogels, respectively and a nozzle’s diameter of 0.254 mm. For this purpose, the parameters of uniformity factor (U), expansion ratio (α), size accuracy, and squareness (Equations (8), (9), (10), and (11), respectively) were calculated using the images taken with the microscope Nikon AZ100 Multizoom (Tokyo, Japan).

Uniformity factor (U) is defined as the difference between the length of the printed structures (l) and the theoretical length (L):

(8) $$U\;=\;\frac{l}{L}$$

The expansion ratio (α) indicates the ability of the ink to spread over the printed surface. It is defined as the relation between the diameter of the printed filament (d) and the theoretical diameter of the nozzle (D):

(9) $$\alpha =\frac{d}{D}$$

Size accuracy quantifies the printing fidelity by comparing the theoretical area (A_t_) (25 mm^2^) of the pores of the designed structure, with the real area (A) of the printed squares [37]:

(10) $$Size\;accuracy=1-\frac{A_{t}-A}{A_{t}}$$

Squareness of the printed scaffolds was also calculated with the perimeter of the pore (L) and the area of the printed square scaffolds (A) [38]:

(11) $$Squareness=\frac{L^{2}}{16A}$$

### 2.6. Cell Culture and Immunofluorescence

In order to adhere the hydrogels for easy handling in the culture place, the surface of coverslips (13 mm in diameter) after washing with ethanol and water was functionalized in a UV-ozone oven with -OH groups. Then, they were immersed in a 10% APTES solution in absolute ethanol for 1 h at 50 °C and subsequently washed with absolute ethanol (×3), Milli-Q water (×2), and PBS (×1). They were then immersed in a solution of glutaraldehyde 2.5% in PBS for 1 h at room temperature, washed with PBS (×3) and Milli-Q water (×3), and finally dried and kept at 4 °C until use.

For cell culture on hydrogels, the corresponding materials were deposited on a slide, and then the glutaraldehyde-functionalized coverslips were placed on top, with UV light used for photo-polymerization and adhesion. Then, Human Bone Marrow Mesenchymal Stem Cells (BM-MSCs) were cultured in DMEM medium (Gibco) supplemented with 10% (*v*/*v*) fetal bovine serum (FBS, Gibco), 1% (*v*/*v*) penicillin/streptomycin (Pen/Strep, Gibco) and 1% (*v*/*v*) GlutaMAX (Gibco) at 37 °C in a humidified atmosphere of 5% CO_2_.

To analyze the adhesion of BM-MSCs to the materials, 10.000 cells were seeded onto photo-crosslinked hydrogels placed in 24-well cell culture plates. 24 h after cell seeding, samples were washed with PBS and chemically fixed using 4% formaldehyde. Next, samples were stained using NucBlue (ThermoFisher, Waltham, MA, USA) and ActinRed (ThermoFisher, Waltham, MA, USA), and fluorescence images were acquired using a Nikon Wclipse Ti DS-Qi2 (Tokyo, Japan) at ×10 fluorescence microscope. Images were processed with ImageJ software.

### 2.7. Cytotoxicity Assay of the “Medium”

Hydrogels were incubated in complete culture medium for 48 h at 37 °C. Simultaneously, BM-MSCs cells were seeded on a 24-well plate at a density of 10.000 cells/well and allowed to adhere for 24 h. Next, half of the culture medium volume of each well was replaced with the hydrogel-conditioned medium, and cells were then cultured for 24 h. Cells were then stained with NucBlue (Thermo Fisher, Waltham, MA, USA) and Live/Dead assay (Invitrogen, Carlsbad, CA, USA) following the manufacturer’s protocol. Finally, five images of each well were acquired using a Nikon TiU (Tokyo, Japan) inverted fluorescence microscope.

## 3. Results

### 3.1. Hydrogel Preparation and Morphological Characterization

The modification of the three biopolymers was carried out via methacrylic acid-mediated methacrylation to introduce active groups for subsequent photo-crosslinking. Nuclear magnetic resonance (^1^H-NMR) confirmed the successful methacrylation of the three polymers.

**Figure 1:**
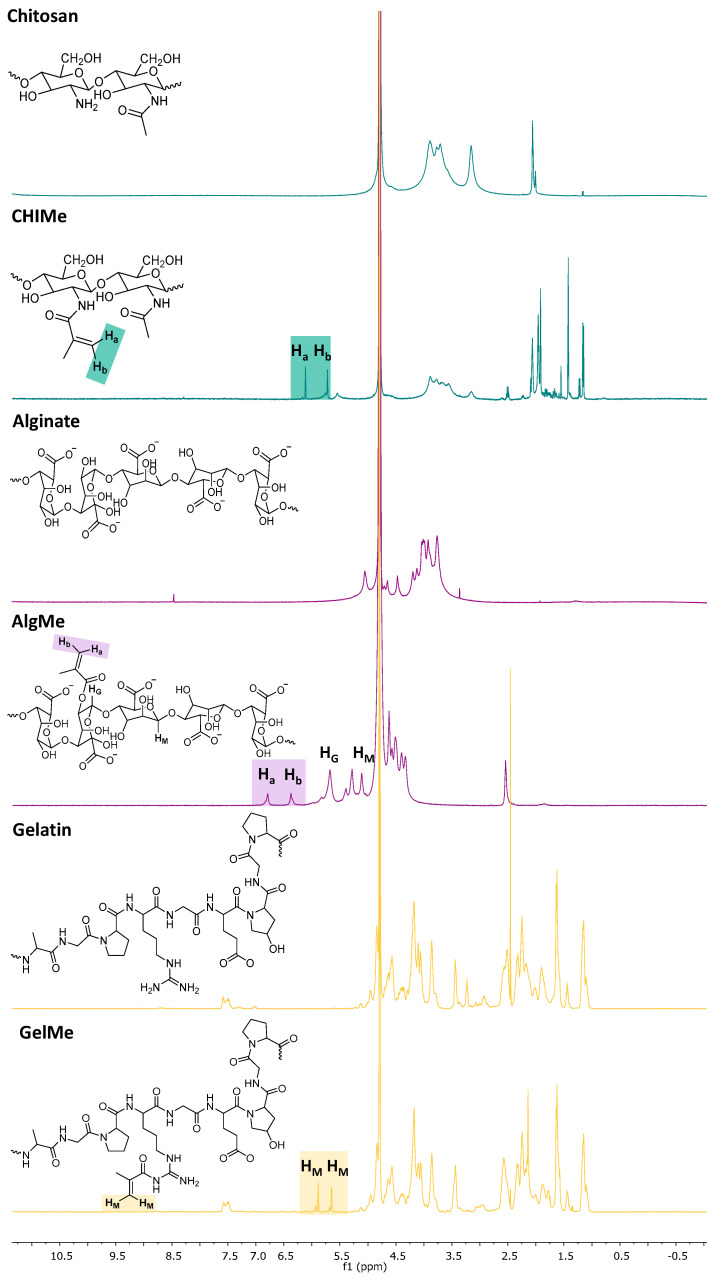
^1^H-NMR spectra of chitosan and methacrylated chitosan (green), alginate and methacrylated alginate (purple), and gelatin and methacrylated gelatin (orange).

Figure 1 compares the ^1^H-NMR spectra of chitosan and methacrylated chitosan. As can be observed, the characteristic signals of the protons of the glucosamine ring appearing in the range of 2.8 and 3.9 ppm decreased in the modified polymer, while two distinct singlets at 5.6 and 6.2 ppm corresponding to the vinyl hydrogens (H_a_ and H_b_) of the methacrylate groups appeared, confirming the successful methacrylation. According to Equation (1), the degree of methacrylation was determined to be 41 ± 4%.

In Figure 1, the ^1^H-NMR spectra of alginate and methacrylated alginate display the expected signals between 3.5 and 4.5 ppm corresponding to the saccharide units of alginate. After modification, additional peaks emerge between 5.7 and 6.2 ppm, attributed to the vinyl protons (H_a_ and H_b_) of the methacrylate groups. These peaks are absent in the unmodified alginate spectrum, confirming the successful incorporation of methacrylate moieties in the alginate structure. The G proportion and the methacrylation degree were calculated according to Equations (2) and (3), resulting in a G proportion of 27± 4% and a methacrylation degree of 9 ± 4%.

The ^1^H-NMR spectra of gelatin and methacrylated gelatin are also shown in Figure 1. The appearance of new peaks between 5.6 and 4.7 ppm (H_M_) confirms the introduction of methacrylate groups in this biopolymer. The signal with the highest chemical shift gives the methacrylate amount present in the gelatin derivatives, while the signal with the smallest chemical shift was not used for quantifications because it was caused by a mixture of the methacrylamide groups and methacryl-modified hydroxyproline [22]. The degree of methacrylation for gelatin derivatives was quantified (32 ± 3%) based on the integration of the H_M_ peak according to Equation (4).

A significantly lower methacrylation degree was measured in the case of the functionalization of alginate due to the fact that only G units are modified in this polymer [21]. In addition, the nucleophilic attack of the carbonyl group of methacrylic anhydride in alginate takes place on -OH moieties, which display a lower nucleophilic nature than the -NH_2_ moieties involved in the functionalization of gelatin and chitosan.

**Figure 2:**
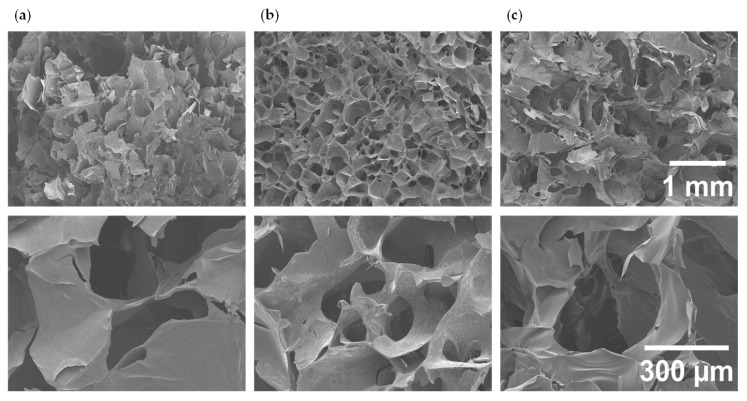
SEM images of (**a**) AlgMe; (**b**) GelMe, and (**c**) CHIMe hydrogels. At the top scale bar = 1 mm and at the bottom scale bar = 300 µm.

After successful methacrylation of the three biopolymers, photo-crosslinked hydrogels were prepared under the conditions described in the experimental section. The measured sol-fraction in all the samples was lower than 10% (CHIMe 7.5 ± 1.1, AlgMe 10.1 ± 0.3 and GelMe 8.3 ± 0.2). Taking into account the importance of porosity in terms of interaction with surrounding cells and tissues, affecting also oxygen and nutrient diffusion, cell migration and proliferation [39], the pore size of the hydrogels was analyzed by scanning electron microscope (SEM). SEM images confirmed that photo-crosslinking leads to interconnected porous three-dimensional structures (Figure 2), with average pore sizes of 0.37 ± 0.07 mm, 0.23 ± 0.08 mm, and 0.13 ± 0.02 mm, for AlgMe, CHIMe and GelMe, respectively. The largest pore size corresponds to AlgMe hydrogels, which is in line with the low methacrylation degree of this polymer, which seems to lead to a lower crosslinking density of the photo-cured network. In the cases of CHIMe and GelMe hydrogels, which display similar degrees of methacrylation (41% and 33%, respectively), the higher concentration of GelMe in the precursor hydrogel mixture (8% GelMe and 1.5% CHIMe) seems to be translated into a higher crosslinking density and, therefore, a smaller pore size.

### 3.2. Physico-Chemical Characterization

**Figure 3:**
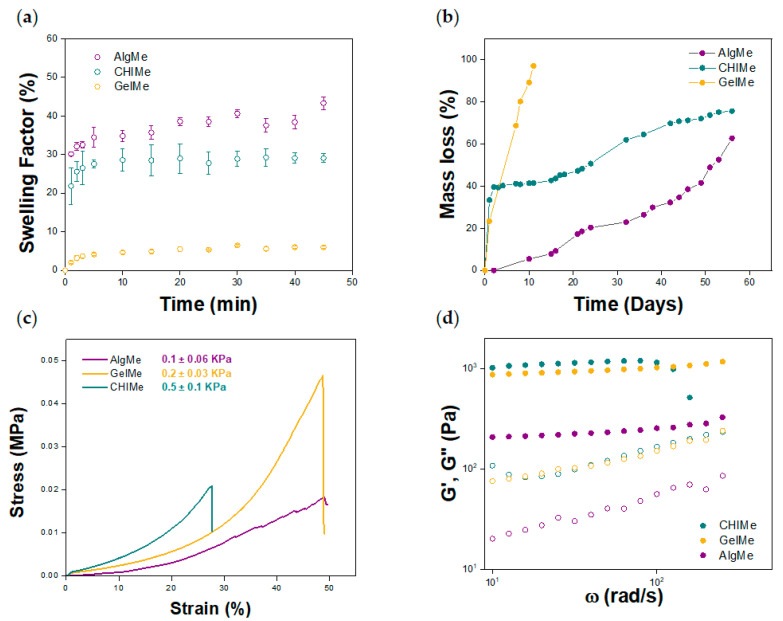
Physico-chemical characterization of the hydrogels. (**a**) Swelling factor; (**b**) Degradation of hydrogels over a period of 60 days; (**c**) Compression stress–strain test, and (**d**) Rheological properties o, CHIMe (green), AlgMe (purple), and GelMe (yellow) in a frequency scan being G’ the storage modulus (filled circles) and G” the loss modulus (open circles).

The porous structure of the hydrogels allows them to absorb and retain water, influencing their mechanical properties and degradation rate. Figure 3a shows the swelling factors of the three hydrogels over time, calculated according to Equation (5). The water absorption capacity is a balance between the pore size of the polymer network, the concentration of the polymer, its methacrylation degree, and its own hydrophilicity. As shown in Figure 3a, in this study AlgMe exhibited the highest swelling factor (43%), which was attributed to its low concentration and methacrylation degree, resulting in a lower degree of crosslinking and consequently a higher water absorption capacity. This is in consonance with the high pore size measured by SEM (see Figure 2a). The considerable water absorption of AlgMe gels is also explained by the chemical nature of the biopolymer, which contains many carboxylate groups that at neutral pH are deprotonated, leading to repulsive electrostatic interactions and promoting higher water absorption by osmotic pressure, compared to gelatin and chitosan, which present more hydrophobic regions after methacrylation and higher crosslinking degrees. The data obtained are in agreement with those reported in the literature [10,40,41]. Regarding CHIMe and GelMe, their swelling factors are in accordance with their corresponding pore sizes measured by SEM. The higher concentration of GelMe that results in a higher crosslinking density leads to a more restricted swelling of GelMe hydrogels.

The kinetics of degradation of the hydrogels is a critical parameter as it directly influences the material’s stability and, subsequently, its potential applicability. Figure 3b shows the hydrolytic degradation under physiological conditions (pH = 7.4) of the three hydrogels after reaching their maximum swelling. Figure 3b shows that the polymer composition governs the hydrogel degradation kinetics rather than the water content (swelling) of the networks. CHIMe achieved a degradation rate of 78% in a hydrolytic medium in 60 days of study. It is known that at pH values higher than its pK_a_ (6.5), chitosan establishes inter- and intramolecular hydrogen bonds due to the protonation of its amine groups, which increases hydrogel stability and consequently reduces its degradation rate [42].

As expected, the slowest degradation rate was obtained for AlgMe, reaching 72% mass loss in 60, which is consistent with reports in the literature [43,44]. The numerous carboxyl and hydroxyl groups on the molecular backbone of alginate can easily form intra- and intermolecular hydrogen bonds that reinforce the molecular network and limit chain mobility. This fact restricts the hydrolytic cleavage of the glycosidic bonds at physiological pH and makes this biopolymer degrade more slowly [45]. Finally, GelMe hydrogels showed the fastest degradation rate, reaching 100% mass loss in 18 days. Gelatin is thermally unstable at 37 °C, presents more disordered conformations, losing its structural integrity, which contributes to accelerating degradation via hydrolysis under physiological conditions [46,47].

Figure 3c shows the compression stress–strain curves measured for the prepared hydrogels. The results obtained indicate that the Young’s modulus increases as the strain increases, showing the characteristic non-linear behavior of hydrogels across all samples [10]. Analyzing the Young’s modulus, it can be observed that AlgMe is the softest hydrogel (lower Young’s modulus), and therefore the most flexible and the one that withstands the highest stress and deformation until breakage. In the case of CHIMe and GelMe hydrogels, which showed more restricted swelling capacity, tougher hydrogels were obtained. Unlike swelling, which seems to be governed by the different concentrations of the polymer in the network, their mechanical behavior seems to be primarily influenced by differences in crosslinking density, as reflected in their degree of methacrylation. A higher degree of crosslinking leads to more rigid and fragile structures, especially in these hydrogels, in which the direct ester bonding between polymeric chains takes place in the absence of an intermediate and flexible crosslinking agent. Thus, CHIMe was the hardest and the most fragile material tested, suffering failure at 27% of compression. In comparison to previously reported data [14], the higher light intensity employed during the photo-printing of the ink (19 mW/cm^2^ vs 0.2 mW/cm^2^) in the present work, makes CHIMe gels capable of withstanding deformation forces 5 times greater.

Rheological tests were carried out to characterize the viscoelastic behavior of the photo-crosslinked hydrogels by measuring the energy stored (storage modulus, G’) in the material during shear and the energy loss afterwards (loss modulus, G’’). The shear stress was controlled by varying the oscillation amplitude. The results obtained are shown in Figure 3d. The rheological results suggest that all samples show the typical hydrogel behavior, as the storage moduli values are higher than the loss moduli in a frequency range (G’ > G’’) of 0.1 to 500 rad/s at a fixed strain of 1%, indicating that the materials exhibit predominantly elastic properties rather than viscous. In addition, G’ presents a direct dependence on the composition of the hydrogel [48]. It is observed that hydrogels with higher swelling capacity (AlgMe), due to their lower crosslinking density, showed reduced G’ values. This indicates that AlgMe-based hydrogels are the most elastic ones, which is in agreement with the data obtained in the analyses of the mechanical properties. On the other hand, hydrogels based on GelMe and CHIMe show higher values of both storage and loss modulus, derived from their higher crosslinking density and higher degree of methacrylation.

### 3.3. Printability

Hydrogels are excellent candidates as matrices for 3D printing. For the purpose of studying the quality of the printing of the prepared inks, square grids were printed following the conditions described in the experimental part, and the uniformity factor expansion ratio, size accuracy and squareness were measured according to Equations (8), (9), (10), and (11), respectively.

**Figure 4:**
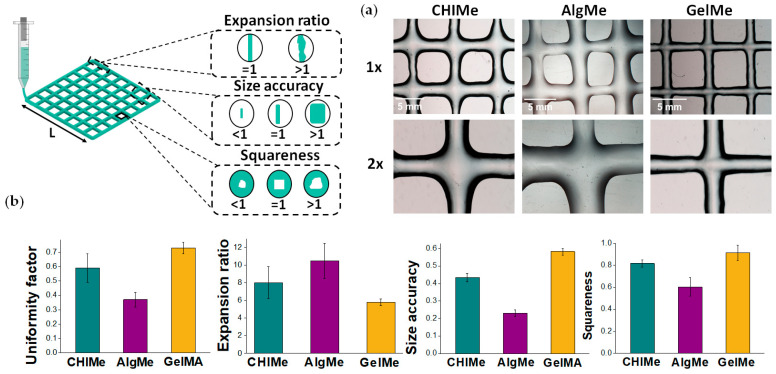
(**a**) Optical microscope photographs of the obtained square scaffolds: CHIMe, AlgMe and GelMe at 1× and 2×. The selected scale is in the upper left section; (**b**) Results obtained from uniformity factor, expansion ratio, squareness, and size accuracy for the three systems tested for 3D printing by extrusion, CHIMe (green), AlgMe (purple), and GelMe (yellow). The data shown represents the average ± standard deviation of the images analyzed.

According to Figure 4, it can be concluded that a high concentration of biopolymer and a high methacrylation degree in the hydrogels lead to a clear improvement of the printability of the ink, increasing the uniformity factor, size accuracy, and squareness, while decreasing the expansion ratio. It should be noted that the methacrylation degree and polymer concentration differ among the studied systems. These values were selected to yield printable, stable hydrogels for each polymer rather than to isolate specific physicochemical parameters. The selected conditions fall within experimentally and commercially relevant ranges. Thus, in the case of GelMe, high concentration and high methacrylation degree lead to a significant improvement in the printability of the ink, attaining uniformity values close to 1. Regarding the expansion ratio, as can be observed in Figure 4a, GelMe filaments are the most stable and thinnest, and consequently, the lowest value is obtained for GelMe ink, while AlgMe value deviates the most. Size accuracy and squareness are also key parameters for evaluating the printability of hydrogels. The obtained results reinforce that AlgMe is the least suitable ink for light-induced extrusion-based 3D printing, as its printed grids exhibit significant heterogeneity and irregularity. The pronounced deviation from the ideal value of 1 in both size accuracy and squareness of AlgMe suggests poor structural stability and filament control during printing, ascribed to its lower mechanical and rheological properties (Figure 3c,d) derived from its low crosslinking density. In contrast, GelMe demonstrates better printability, yielding grids with greater uniformity and closer adherence to the expected square geometry. These findings highlight the impact of hydrogel composition and elastic properties on print resolution and structural integrity, emphasizing the limitations of the employed AlgMe solution in achieving precise and regular scaffolds. This limitation of AlgMe is consistent with our previous results, in which a highly concentrated methacrylated alginate solution was found to be unprintable when additional vinyl crosslinking agents were not incorporated into the formulation [49].

### 3.4. Biocompatibility and Analysis of Cell Attachment

**Figure 5:**
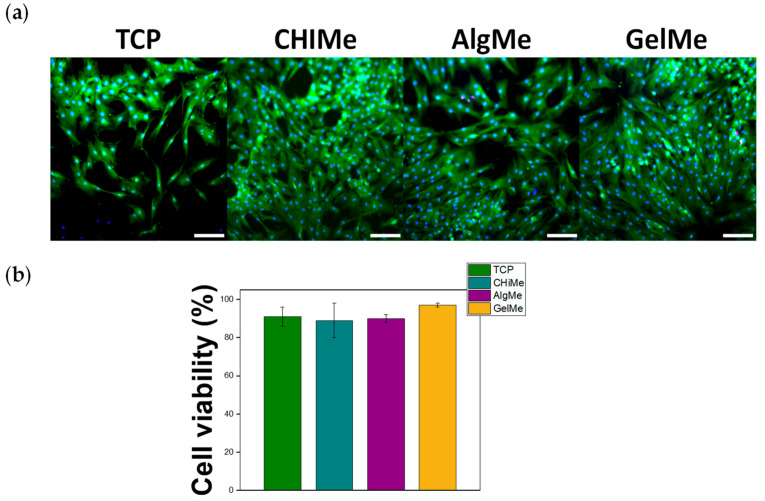
(**a**) Representative fluorescent images of BM-MSCs cultured with the indicated conditioned media for 1 day. Blue shows nuclei, red dead cells, and green live cells; (**b**) Quantification of the Live/Dead assay, n = 3. Scale bar = 200 µm.

Cell viability assay was performed to confirm that the degradation products of these hydrogels could provide a biocompatible environment for cells during the culture process. The cell viability of BM-MSCs cells cultured on the medium where CHIMe, AlgMe and GelMe hydrogels had remained in their initial degradation stage, was not significantly different from cells cultured on tissue culture plastic (TCP). This indicates that not only do the hydrogels display good cytocompatibility, as has been commonly proven in previous studies, but the degradation medium of these hydrogels is also cytocompatible (Figure 5).

**Figure 6:**
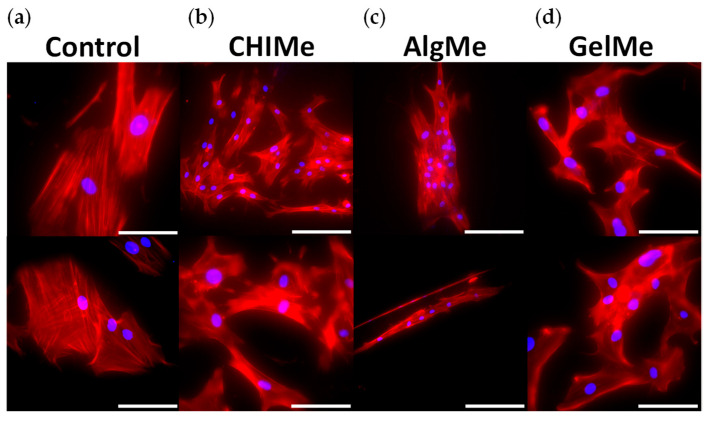
(**a**) Fluorescence images of nuclei (blue) and phalloidin (red) corresponding to BMSCs seeded on Control (coverslips); (**b**) CHIMe; (**c**) AlgMe, and (**d**) GelMe hydrogels. Scale bar = 200 µm.

Cell adhesion, spreading, and cytoskeleton organization are important parameters for assessing cell compatibility and the suitability of biomaterials for a required application [50]. Cell adhesion is affected by surface wettability, structure, and surface chemical composition on the scaffold. These factors also influence cell proliferation, migration, and physiological response [51]. Taking this into account, we analyzed the early response of BM-MSCs adhering to hydrogels by immunofluorescent staining of actin cytoskeleton and nuclei. The results obtained are shown in Figure 6.

Our results indicate that BM-MSCs cells adhere to the three hydrogels, but as expected, the spreading area of the cells was significantly larger on the GelMe hydrogels. This is a consequence of the fact that gelatin is hydrolyzed collagen, one of the major components of the ECM, and displays cellular adhesion points, which facilitate the adhesion process. In addition, the GelMe network showed the smallest pore size in SEM analyses, which provides a larger surface area for cell adhesion [52]. However, alginate hydrogels exhibit the lowest cell adhesion, which may be attributed to their well-known poorer ability to interact with cells [53] due to their surface charge, as well as the lower crosslinking density, leading to lower stiffness and higher pore dimensions [54].

## 4. Conclusions

This study presents and comparatively analyzes three well-known photo-crosslinkable hydrogel formulations based on naturally derived biopolymers—chitosan, alginate, and gelatin—aimed at mimicking the ECM for tissue engineering applications. The methacrylation process was confirmed via NMR. The morphological analysis of photo-crosslinked hydrogels demonstrated the formation of an interconnected porous structure, and the physicochemical characterization revealed that swelling capacity, degradation rate, and mechanical behavior were strongly influenced by polymer concentration and crosslinking density. Rheological studies confirmed that all hydrogels exhibit predominantly elastic behavior, with CHiMe and GelMe presenting the highest elastic modulus and thus, the highest structural integrity and print fidelity in extrusion-based 3D printing. Additionally, biocompatibility assessments confirmed that all formulations supported cell viability, with GelMe promoting better cell adhesion. Table 1 summarizes the comparative performances of the three studied systems. These findings suggest that GelMe is the most promising candidate for extrusion-based 3D bioprinting, in comparison to the AlgMe and CHIMe inks presented here.

## Figures and Tables

**Table 1:** Comparative analyses of the main physico-chemical properties, printability, and cell viability of the studied photo-crosslinkable hydrogels.

		GelMe	AlgMe	CHIMe
MD (%)		32 ± 3	9 ± 4	41 ± 4
Swelling (%) after 45 min.		6 ± 0.2	38 ± 8.7	29 ± 1.1
Time for 50% of mass loss (days)		5	52	24
Young Modulus (KPa)		0.20 ± 0.03	0.10 ± 0.06	0.50 ± 0.10
Cell viability (%)		97 ± 1	90 ± 2	89 ± 9
Printability	Uniformity Factor	☑☑☑	☑	☑☑
	Squareness	☑☑☑	☑	☑☑

☑ low; ☑☑ intermediate; ☑☑☑ high.

## Data Availability

The original contributions presented in this study are included in the article. Further inquiries can be directed to the corresponding author.
